# 

**Published:** 1894-03

**Authors:** 


### CONTENTS.

*EDITORIALS.*

| | PAGE |
|---|---|
| A Word with our Subscribers, | 65 |
| The Warning of Constantine Hering, | 65 |
| The New York Homœopathic Union, | 69 |

*MISCELLANEOUS.*

| | |
|---|---|
| Proceedings of the New York Homœopathic Union, | 69 |
| The Antidote to Carbolic Acid. E. Carleton, M. D., | 77 |
| Metastasis of Gonorrhœa. I. Dever, M. D., | 80 |
| Tuberculinum and Isopathy. A. W. Holcombe, M. D., | 82 |
| A "Refuse" Case. J. B. Campbell, M. D., | 83 |
| The Jubilee of the American Institute, | 84 |
| A Result of Vaccination, | 85 |
| Gleanings. F. H. Lutze, M. D., | 86 |

*BOOK NOTICES.*

| | |
|---|---|
| Establishing a New Method of Artificial Respiration in Asphyxia Neonatorum, | 92 |
| Proceedings at the Formal Opening of the Engineering Building of the Pennsylvania State College, | 93 |
| The Strike at Shane's, | 93 |
| A New Unabridged Pronouncing Dictionary of Medicine, | 94 |
| Childhood, | 94 |
| Vick's Floral Guide, 1894, | 94 |
| A System of Legal Medicine, | 95 |
| A Text-book of the Theory and Practice of Medicine by American Teachers, | 95 |

*NOTES AND NOTICES.*

| | |
|---|---|
| The Missouri Institute of Homœopathy—E. B. Treat—A Manual of Clinical Diagnosis—Diseases of Hair and Scalp—How to Use Forceps—Copartnership Notice, | 96 |

